# What is known about pressure injury prevention and management approaches among individuals with mobility impairments participating in disability sport? A scoping review

**DOI:** 10.1111/1440-1630.70128

**Published:** 2026-10-08

**Authors:** Callum Parker, Sjaan Gomersall, Emma Beckman, Sean Tweedy, Jessica Hill

**Affiliations:** ^1^ School of Health and Rehabilitation Sciences The University of Queensland Brisbane Queensland Australia; ^2^ School of Human Movement and Nutrition Sciences, Health and Wellbeing Centre for Research Innovation The University of Queensland Brisbane Queensland Australia; ^3^ School of Human Movement and Nutrition Sciences The University of Queensland Brisbane Queensland Australia

**Keywords:** adaptive sports, decubitus, para sport, persons with disabilities, pressure ulcer, wheelchair sport, wound care

## Abstract

**Introduction:**

Pressure injuries are a significant health concern for individuals with mobility impairments, impacting participation and quality of life. Sport is a meaningful leisure occupation for many but poses unique occupational, physical, and environmental challenges which increase the risk of pressure injuries. Occupational therapists can support those with mobility impairments in balancing pressure injury risk and participation in sport; however, little is known about effective management in sport. This scoping review aimed to map existing evidence on pressure injury prevention and management approaches among individuals with mobility impairments participating in disability sports.

**Methods:**

A systematic search of six databases, grey literature, policy documents, and guidelines was conducted in August 2025. Sources were included if they addressed individuals of any age, participating in sports using adaptive equipment and reported prevention or management approaches for pressure injuries. Findings were synthesised using the International Classification of Functioning, Disability and Health (ICF) framework.

**Consumer and community involvement:**

Consumers and community members were not involved in this scoping review.

**Results:**

Ten sources met inclusion criteria, with representation from eight disability sports: seven quantitative studies, one mixed methods study, one position statement, and one clinical guideline. Owing to the immaturity of the field, both the clinical guideline and position statement drew on minimal evidence specific to sport, limiting their applicability.

**Conclusion:**

Evidence on pressure injury prevention and management in disability sport is largely focused on the body functions and structures domain, with limited consideration for activities, participation, and environmental factors that influence risk. The evidence base remains underdeveloped, limiting occupational therapists' access to evidence‐informed strategies for pressure injury prevention and management in this population. Future research should prioritise occupational analyses of sports participation, mapping tasks, environments, and equipment interfaces across different sports and athlete groups. Generating sport‐specific evidence will enable robust guidelines and occupation‐based interventions that align with athletes' real‐world experiences.

## PLAIN LANGUAGE SUMMARY

Pressure injuries are sores that can happen when prolonged pressure from equipment such as sport wheelchairs damage the skin and underlying tissue. They are a serious health problem and can cause pain, infection, and long periods of bed rest. Disability sports often involve using special equipment, like sport‐specific wheelchairs, that might not fit properly and have harder surfaces which increase the risk of a pressure injury. Currently, there is very little research or guidelines for occupational therapists about how to manage these risks in sports. This review examined existing research on preventing and managing pressure injuries for people with mobility impairments who play sports. We included research about people of any age, who played sports using adaptive seating and reported ways to prevent or manage these injuries. We found 10 sources which mostly focused on adding more supportive cushions to the sports equipment or changing the way a person sits in the wheelchair to reduce pressure. One study asked coaches and athletes how much they knew about managing pressure injuries and their knowledge was limited. The two guidelines we found included limited sports‐specific research to inform their recommendations. More research is needed to create better quality, evidence‐based guidelines for occupational therapists to support people with disabilities to safely play sports.

**Key Points for Occupational Therapy**

- Occupational therapists are uniquely positioned to support participation in disability sport through occupation‐based risk assessment and intervention.
- The evidence base for pressure injury prevention and management in disability sports is underdeveloped.
- Current guidelines have minimal sport‐specific evidence guiding recommendations and rely on the general literature.

## 1 INTRODUCTION

Sport is an important occupation for many Australians and is widely recognised for its health and wellbeing benefits (Eather et al., 2023; Oja et al., 2024). Disability sport is the collective term describing sporting activities that are adapted or purposefully designed for people with disabilities (Sportanddev, 2024). Similar to participation in sport among people without disabilities, engagement in disability sport is associated with positive physical and psychosocial outcomes for individuals with mobility impairments (Sportanddev, 2024). Research evidence highlights gains in strength, balance, pulmonary function, and ease of transfer (Rayes et al., 2022), as well as supporting psychosocial wellbeing, with individuals reporting benefits to self‐esteem and overall quality of life (Watson et al., 2023). These benefits have driven increasing interest in disability sport globally.

Interest in disability sports continues to grow, in part driven by the International Paralympic Committee's commitment to increase opportunities for persons with disabilities to participate in sports at all levels (International Paralympic Committee, 2025). In 2032, Brisbane will host the Olympic and Paralympic Games. Australia's bid to host these two large‐scale events included a commitment to increase the number of people with disabilities participating in sport by 500,000 (Australian Olympic Committee, 2021).

Of the 5.5 million Australians with a disability, approximately 16%, or one in seven, identify as using a mobility aid, with over 180,000 requiring a wheelchair (Australian Bureau of Statistics, 2022; Goldsmith, 2023). For people with disabilities, particularly for those with mobility and sensory impairments, who are at higher risk of pressure injuries (i.e., wheelchair users and amputees), there are unique barriers to participation. A pressure injury (also known as pressure ulcer) is damage to the skin, typically over a bony prominence, caused by intense and/or prolonged pressure or pressure combined with shear, resulting in tissue necrosis (European Pressure Ulcer Advisory Panel et al., 2019). Pressure injuries are of particular concern if not managed appropriately, as the high risk for infection can lead to sepsis, multisystem organ failure, and death (Zhu et al., 2025). Pressure injury development is influenced by a number of personal factors, including skin and nutritional status, with poor perfusion and immobility increasing risk (Chung et al., 2022). Pressure injuries are also influenced by external factors such as the ergonomics and fit of adaptive equipment, temperature, and presence of moisture, of particular relevance in sport (Chung et al., 2022). Within sporting contexts, the use of specialised equipment, such as sports wheelchairs and prostheses, may compromise skin integrity due to poor fit, design prioritising performance over pressure relief, and increased pressure exerted while completing complex, physical movements (Maeda et al., 2021). Long‐haul travel, common in elite sport, further exacerbates risk due to prolonged sitting, with limited access to appropriate seating and usual healthcare (Ay Yıldız et al., 2025). Indeed, pressure injury development is not limited to elite athletes; as in grassroots adaptive sports, there is often limited access to appropriately fitted equipment. While there is limited literature exploring the risk factors for pressure injuries for people with disability participating in grassroots sport, pressure injury literature indicates that poorly fitting equipment significantly influences the risk of pressure injury (Vecin & Gater, 2022).

Recent research has found that paralympic athletes report developing pressure injuries on average three times per year (Ay Yıldız et al., 2025), with skin issues being reported as one of the most common injuries for individuals participating in disability sports (Patatoukas et al., 2011). Evidence from sporting contexts points to substantial pressure injury risk; however, research largely targets elite athletes, with minimal consideration for those entering sport (Mutsuzaki et al., 2014; Shimizu et al., 2017; Tamai et al., 2020). Various systematic reviews estimate pressure injury incidence to be between 23% and 33% outside of sports (Chen et al., 2020; Shiferaw et al., 2020). In contrast, Shimizu et al. (2017) and Mutsuzaki et al. (2014) found that 45%–60% of elite wheelchair basketball players presented with deep tissue injuries, a type of pressure injury characterised by damage to the underlying tissue, which is hypothesised to be the origin of most pressure injuries (Berlowitz & Brienza, 2007). Beyond the acute physical risks, pressure injuries have the potential for negative psychological and social impacts. Current best practice guidelines for managing pressure injuries recommend offloading affected areas, often involving long periods of bedrest (European Pressure Ulcer Advisory Panel et al., 2019). This significantly restricts engagement in meaningful occupations, increasing social isolation, and contributing to accelerated physical deconditioning (Felsenberg et al., 2009) and poor mental health outcomes, a major concern among persons with disabilities (Liu et al., 2025).

Sports participation is a meaningful leisure occupation that supports wellbeing, inclusion, and identity development (Law et al., 1996). As occupation is a primary means by which identity is constructed and expressed, disability sport can play a significant role in the formation of occupational identity (Hansson et al., 2022). When a pressure injury forces an athlete to withdraw from training or competition, the consequences extend well beyond the physical wound. The resulting occupational disruption, characterised by enforced periods of bedrest and restricted activity, constitutes a form of occupational deprivation, the state in which a person is prevented from engaging in occupations that are meaningful and necessary to their wellbeing (Whiteford, 2000). This highlights that pressure injury prevention in sport is not solely a biomedical or biomechanical concern; it is an occupational justice issue, with implications for athletes' right to safe and equitable participation in the occupation of sport.

This framing positions occupational therapists as central contributors to pressure injury prevention in sport. Rather than just equipment assessors, occupational therapists are clinicians who address the full occupational context of risk. Occupational therapists are frequently involved in the assessment and management of pressure injuries (Slayton et al., 2021) and are uniquely positioned to support athletes through occupation‐based risk assessment and intervention that extends well beyond equipment prescription. The International Classification of Functioning, Disability and Health (ICF) framework provides occupational therapists with a structured lens through which to analyse disability sport, enabling examination of how body functions and structures, activity and participation, and sport‐specific contextual factors dynamically interact to shape athletes' occupational performance (World Health Organization, 2001). This analysis enables occupational therapists to identify how the unique demands of the sport intersect with individuals' risk factors in ways that general clinical assessment tools are not designed to capture.

Evidence‐based practice is a core competency of occupational therapy, essential to safe and effective clinical care (Occupational Therapy Board of Australia, 2018). When assessing for risk of pressure injury outside of the sports context, occupational therapists use clinical guidelines which provide recommendations, used alongside clinical judgement, to make informed decisions about prevention and management (European Pressure Ulcer Advisory Panel et al., 2019). However, current risk assessments for pressure injury prevention, one of the most effective ways to reduce their burden, have little consideration for physical activity and sports participation, activities that can have a significant impact on skin integrity. Occupational therapists are required to make critical decisions on prevention and management; however, there is uncertainty on the breadth and strength of evidence available to inform decision making (Serafin et al., 2025).

Despite the critical role occupational therapists can provide in pressure care management (Macens et al., 2011), their role within sports contexts remains poorly defined and inconsistently integrated (Bulan et al., 2025). The lack of occupational therapy involvement is multifaceted. Funding constraints, a lack of specialisation, and no available synthesis of the evidence limit the impact occupational therapists can have on supporting safe sports participation through managing pressure injury risk (Bulan et al., 2025). In the absence of targeted prevention and management strategies, individuals engaged in disability sports are at elevated risk for pressure injuries and associated adverse health outcomes.

Disability sport is an emerging area of research with growing global participation; however, pressure injuries continue to limit participation and performance. Despite this, there has been no synthesis of the prevention and management strategies currently used in sport, restricting clinicians' ability to provide evidence‐informed care. Given the well‐established benefits of sport for people with disabilities and the elevated risks associated with participation, there is a clear need for sport‐specific, evidence‐based approaches to pressure injury prevention and management. To address this gap, we conducted a scoping review to map existing research and policy documents and identify priorities for future research with findings organised using the ICF framework (World Health Organization, 2001), providing a structure well suited to examining the multifactorial nature of pressure injury risk in sport. This review aimed to address the following research questions: (1) What is known about pressure injury prevention and management approaches among individuals with mobility impairment participating in disability sports? and (2) What is the reported effectiveness of pressure injury prevention and management approaches among individuals with mobility impairments participating in disability sports?

## 2 METHODS

### 2.1 Study design

A scoping review was determined to be the most suitable methodology as it supports the broad exploration of diverse evidence sources to determine the size and scope of available literature (Grant & Booth, 2009). This approach was particularly appropriate given the limited research available within the sporting context. The scoping review was conducted in accordance with the JBI methodology for scoping reviews (M. Peters, Godfrey, et al., 2024). The review protocol was registered in the Open Science Framework (OSF) online platform (https://osf.io/rtfw2) (Foster & Deardorff, 2017).

### 2.2 Search strategy

A four‐step search strategy was utilised in this review.

- A preliminary search of MEDLINE, the Cochrane Database of Systematic Reviews and JBI Evidence Synthesis was conducted in June 2025 to ensure that there were no current or underway systematic reviews or scoping reviews on the topic. None were identified.
- Text words contained in the titles and abstracts of relevant articles, and the index terms used to describe the articles were used to develop a full search strategy, in consultation with a librarian, for the following databases: MEDLINE (Pubmed), Embase (Elsevier), CINAHL (EBSCOHost), Web of Science, Scopus, and SPORTDiscus which was completed in August 2025. Table 1 includes the search strategy for PubMed.
- A Google search was conducted using a systematic search strategy to identify grey literature and policy documents
- The reference lists of all included sources of evidence were screened for additional sources that may not appear in the databases

**TABLE 1:** Search strategy for Pubmed database 9.08.2025.

Search strategy	Results
MEDLINE (PubMed) ("Para‐Athletes"[Mesh] OR "Sports"[Mesh] OR "sport*"[tiab] OR "para‐athlete*"[tiab] OR "para athlete*"[tiab] OR "parathletic*"[tiab] OR "paralympian*"[tiab] OR "paralympic athlete*"[tiab] OR "disabled athlete*"[tiab] OR "parasport*"[tiab] OR "para‐sport*"[tiab] OR (("athlete*"[tiab] OR "sport*"[tiab]) AND ("Persons with Disabilities"[Mesh] OR "disabled"[tiab] OR "disabilit*"[tiab] OR "wheelchair"[tiab]))) AND ("Pressure Ulcer"[Mesh] OR "pressure injur*"[tiab] OR "pressure ulcer*"[tiab] OR "pressure sore*"[tiab] OR "decubitus ulcer*"[tiab] OR "decubitus sore*"[tiab] OR "bedsore*"[tiab] OR "bed sore*"[tiab] OR "pressure area*"[tiab] OR "deep tissue injur*"[tiab])	144

### 2.3 Inclusion criteria

Inclusion criteria were based on the JBI Population, Concept, Context (PCC) framework (M. Peters, Godfrey, et al., 2024). This scoping review included published studies and grey literature available in English with full text available. There were no restrictions on geographical location and no limitations on timeframe of publication. Articles were included if they were peer‐reviewed research papers, including both qualitative and quantitative designs. Grey literature was also included, including unpublished theses and published policy documents and guidelines. Articles included individuals with mobility impairments of all ages participating in sports, at both the elite and recreational levels, and were required to include information about preventing pressure injuries and approaches to the assessment and management of existing pressure injuries. Articles were excluded if they were a review, editorial, conference abstract or protocol. Further, articles were also excluded if they discussed only the incidence of occurrence among individuals with mobility impairments, and not the prevention, assessment, and management of pressure injuries.

### 2.4 Evidence selection

Search results were uploaded into Covidence and duplicates removed. The results were independently screened by two authors (CP and JH), starting with title and abstract. Full text screening was completed independently by the same authors. Disagreements were resolved by discussion between the two authors.

### 2.5 Data extraction

The data extraction table was developed by the author (CP) and piloted by two authors independently (CP and JH). One author completed data extraction (CP), with the extraction table reviewed by a second author on completion (JH). Disagreements that arose between reviewers were resolved through discussion.

### 2.6 Data analysis and presentation

The data gathered from the search were analysed using a descriptive and thematic analysis approach consistent with scoping review methodology. Findings were organised using the ICF framework (World Health Organization, 2001), selected for its capacity to capture the interaction between body functions and structures, activity and participation, and contextual factors relevant to pressure injury risk in sport and its relevance to occupational therapy practice.

### 2.7 Positionality statement

The research team comprised researchers with allied health backgrounds, including occupational therapy, physiotherapy, and clinical exercise physiology. We identify as nondisabled and acknowledge our clinical and academic backgrounds shape how we interpret literature on disability. We adhered to a well‐established systematic scoping review methodology to enhance transparency and minimise bias (M. Peters, Godfrey, et al., 2024).

## 3 RESULTS

After duplicates were removed, 469 sources were identified for title and abstract screening. Following abstract screening, 49 full‐text sources were reviewed, with 10 meeting inclusion criteria. The PRISMA flow diagram is presented in Figure 1.

**FIGURE 1:**
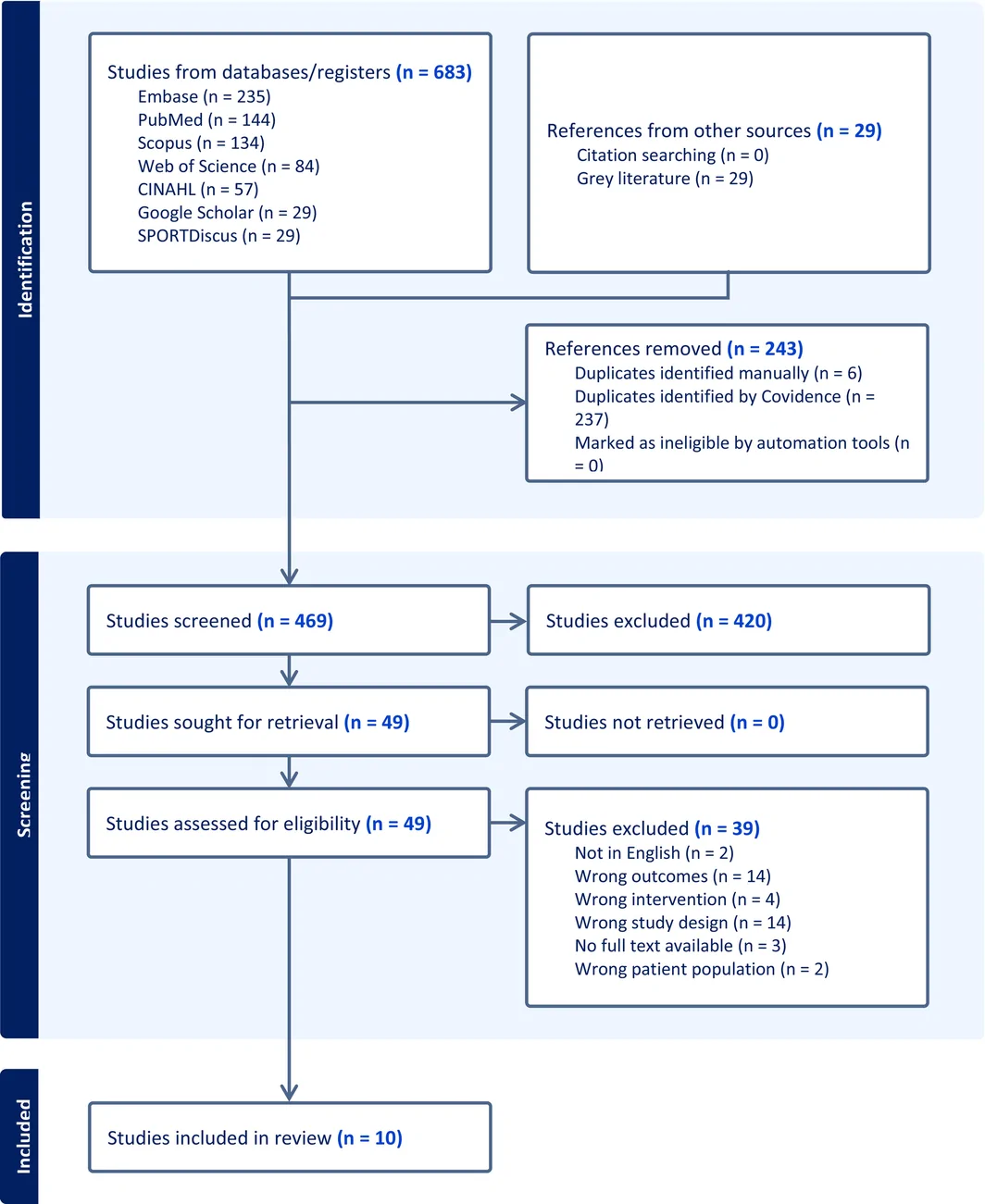
PRISMA flow chart. Alt text: PRISMA flow diagram illustrating study selection: 683 sources identified through database searching, 29 additional records identified through alternate sources, 243 duplicates removed. A total of 469 sources screened, 49 full text sources assessed for eligibility, and 10 sources included in the review.

Included sources comprised seven quantitative studies, one mixed methods study, one clinical practice guideline, and one position statement. A summary of source characteristics is provided in Tables 2 and 3. Findings are organised thematically, according to three domains of the ICF (World Health Organization, 2001): (1) body functions and structures, (2) activity and participation, and (3) contextual and environmental factors. Sources were heavily concentrated in the body functions and structures domain while activity, participation, and contextual dimensions were underrepresented. No included studies directly measured the clinical effectiveness of pressure injury prevention or management in disability sport. Collectively, the evidence base available is insufficient to support conclusions about the effectiveness of any specific prevention or management approach in disability sport.

**TABLE 2:** Study characteristics.

Author(s)	Year of publication	Country of origin	Aims/purpose	Study population and sample Size	Methodology
Berthold et al	2013	USA	(a) To determine if subjects with disabilities have higher average seated pressures than those without disabilities (b) To determine if knee position is related to average seated pressure (c) To determine if cushion use is related to average seated pressure	13 adult ice sledge hockey athletes (meeting Paralympic participation criteria). 13 control subjects (without physical disability).	Quasi‐experimental
Darrah et al	2014	USA	To compare seated pressure distributions between groups and assess risk for pressure injury. Comparing cushion use vs. no cushion use and knee flexion vs extension.	15 ice sledge hockey athletes (meeting Paralympic participation criteria). 15 age matched controls (without physical disability)	Quasi‐experimental
Lewis et al	2017	Australia	To investigate the efficacy of pressure mapping technology in quantifying athlete‐wheelchair interaction at the seating interface, and the influence of foam inserts on pressure.	6 wheelchair racing athletes (4 junior and 2 senior athletes).	Quasi‐experimental
Anderson et al	2018	USA	To investigate whether commercially available cushioned shorts would add skin protection when used in adaptive sports equipment (wheelchair rugby, wheelchair basketball and handcycle).	9 Adults wheelchair users with SCI. 16 able‐bodied participants.	Quasi‐experimental
Rice et al	2018	USA	To explore how wheelchair setup and individual athlete characteristics influence pretibial skin pressures during racing, in both static and dynamic conditions.	11 elite wheelchair racers (T53 and T54) with traumatic SCI or spinal disease.	Cross‐sectional
Tang et al	2019	Singapore	To improve the performance and comfort of para table tennis athletes by enhancing the design of their wheelchairs, focusing on: Increasing sweep area (reach across the table) Reducing pressure‐related discomfort and fatigue Preventing pressure sores during prolonged training and competition	2 para table tennis athletes.	Single case experimental
Peters et al	2020	USA	To examine the influence of personal and wheelchair factors (seat dump angle, backrest height and cushion type) on seat interface pressure and shear at the ischial tuberosities of wheelchair basketball players during stationary and propulsive conditions.	17 adult wheelchair users participating in wheelchair basketball.	Cross‐sectional
Yildiz et al	2025	Turkey	To determine the knowledge and attitudes of physically disabled Paralympic athletes and their coaches about pressure injuries. Improve recognition and management of pressure injuries to minimise their impact on health and performance.	10 paralympic coaches 82 Paralympic athletes	Exploratory sequential mixed design

**TABLE 3:** Practice statement and guideline characteristics.

Author(s)	Publishing organisation	Year of publication	Country of origin	Target population	Aims/purpose
Tweedy et al	Exercise and Sports Science Australia (ESSA)	2017	Australia	People with spinal cord injury participating in exercise and sport.	To provide safe and effective exercise recommendations for people with spinal cord injury
Direen et al	Australian Institute of Sport (AIS)	2025	Australia	Para‐athletes at risk of pressure injury	To provide general recommendations to guide assessment, prevention and management of pressure injuries for athletes in para sports.

### 3.1 Body functions and structures—Interface pressure and equipment‐based approaches

The majority of included sources (seven of eight empirical studies) focused on the body functions and structures domain, examining interface pressure between the athlete and adaptive sports equipment as the primary mechanism of pressure injury risk. All seven studies used pressure mapping technology to measure seated interface pressure, and interventions were focused on reducing pressure magnitude through equipment or positioning changes.

Five experimental studies tested the effect of equipment modifications. In wheelchair racing, adding a cushion to the tibial region was associated with reduced peak pressures (Lewis et al., 2017). In para ice hockey, introducing a cushion showed no significant pressure benefit in two studies; however, greater knee extension was consistently associated with lower seated pressures across both (Berthold et al., 2013; Darrah et al., 2016). Anderson et al. (2018) found highly variable effects on using cushions to influence seated pressure due to the heterogeneity of adaptive equipment trialled and recommended individual pressure mapping prior to any cushion use. Tang et al. (2019) modified wheelchair design for para table tennis players to address lateral stability and reduce injurious postures.

Two observational studies found that seating configuration was associated with pressure outcomes in wheelchair basketball and wheelchair racing, respectively, with greater seat dump angle, backrest height, and therapeutic cushion use being associated with reduced peak pressures (J. Peters et al., 2020). Increased kneeling plate angles were moderately associated with higher dynamic pressures, particularly in athletes with less trunk function (Rice et al., 2019).

### 3.2 Activity and participation—Sport demands, occupational performance, and behavioural factors

A single empirical study addressed the activity and participation domain. Ay Yıldız et al. (2025) surveyed 82 Paralympic athletes and 10 coaches across four sports and conducted semi‐structured interviews to explore knowledge and attitudes toward pressure injury prevention. Findings revealed that both athletes and coaches demonstrated limited and largely non–evidence‐based knowledge of pressure injury prevention using strategies such as massage and ring cushions, neither of which is recommended in current clinical guidelines (European Pressure Ulcer Advisory Panel et al., 2019). Athletes reported that their primary source of pressure injury information was peer experience rather than health professional advice, underscoring a significant gap in structured, athlete‐centred education (Ay Yıldız et al., 2025). Table 4 provides a summary of the studies included in the review.

**TABLE 4:** Summary of study findings.

Author(s)	Methodology	Prevention and/or management approach	Results
Berthold et al. (2013)	Pressure mapping using FSA pressure mapping system in ice hockey sledge seat with no cushion or Vicair all‐rounder cushion with disabled and control (able bodied) group. Mean pressure recorded for: No cushion with knees flexed (45°) Cushion with knees flexed (45°) No cushion with knees extended (180°) Cushion with knees extended (180°)	Prevention approach: Pressure mapping to identify suitable cushions that reduce mean seated pressure. Prevention approach: Pressure mapping to identify suitable body positions that reduce mean seated pressure.	Pressure mapping can provide data to make informed decisions about cushion use and knee position in ice hockey sledges to reduce average seated pressure. However, the authors acknowledged that all disabled athletes exceeded maximum pressure reading (200 mmHg) of the sensor, which may have falsely lowered the readings when cushion was not present. Disability group: No significant differences in average pressure in a knee flexed or extended position. Cushion use had no effect on average pressure. Control group: Average seated pressures were significantly higher when the knees were flexed with a cushion (*p* = 0.011) and without (*p* = 0.011).
Darrah et al (2016)	Pressure mapping using FSA pressure mapping system in ice hockey sledge seat with no cushion or Vicair all‐rounder cushion with disabled and control (age matched able bodied) group. Mean pressure recorded for: No cushion with knees flexed (45°) Cushion with knees flexed (45°) No cushion with knees extended (180°) Cushion with knees extended (180°)	Prevention approach: Pressure mapping to identify suitable body positions that reduce mean seated pressure. Prevention approach: Use of pressure redistribution cushion to reduce average seated pressure.	Pressure mapping can provide data to make informed decisions about cushion use and knee position in ice hockey sledges. However, authors noted that disabled athletes reached sensor saturation (299.95 mmHg) potentially underrepresenting the maximum pressure in this group. Disability group: Cushion had no effect on pressure (*p* = 0.443) Control group: Knee extension showed significantly lower average pressures in controls, both with the cushion (*p* < 0.001) and without (*p* < 0.001)
Lewis et al (2017)	Pressure mapping using XSensor LX100 at 4 Hz for 10s trial. Pressure distribution at the pretibial region measured during propulsion phase of wheelchair racing athletes' performance. Participants measured with and without presence of foam cushion. Peak pressure (PP), average pressure (AP), and contact area (CA) recorded.	Prevention approach: Pressure mapping to identify PP, AP, and CA in propulsion phase of wheelchair racing athletes' performance. Prevention approach: Utilising custom foam cushion inserts at pretibial region to reduce AP, PP, increase CA and enhance seated stability.	Pressure mapping can capture AP, and CA during propulsion phase as it does not interfere with athletes' motion. Unable to measure PP due to sensor reaching saturation (pressure values exceeded the measuring capabilities of the sensor). Foam cushion use showed a significant Decrease in AP and significant increase in contact area (*p* < 0.01).
Anderson et al. (2018)	Pressure mapping using TekScan pressure mapping system. Average pressure (AP) and peak pressure (PP) recorded. Three studies completed: Eight adults mapped in daily wheelchair and adaptive equipment with and without bicycle shorts. Sixteen able‐bodied subjects mapped with and without an impact short and full coverage short in a hand cycle. One adult mapped with and without full coverage shorts in basketball wheelchair and hand cycle.	Prevention approach: Use of cushioned shorts to reduce AP and PP.	Pressure mapping can provide AP and PP data to support cushion selection. Impact shorts increased PP and AP (*p* = 0.00, *p* = 0.00) compared to no shorts (basketball wheelchair and handcycle). Full coverage padded shorts decreased AP (*p* = 0.029) but had no impact on PP (*p* = 0.756) compared to no shorts (basketball wheelchair and handcycle). Static AP and PP was significantly higher in adaptive sports equipment compared with daily wheelchairs, AP (*p* = 0.023) and PP (*p* = 0.052).
Rice et al (2019)	Pressuring mapping using CONFORMat 5330 placed on kneeling plate of racing wheelchair of T53 and T54 athletes while on dynamometer. Peak pressure (PP) and average pressure (AP) measured at 10 Hz over 20‐s trial in ready stance and propulsion at 14mph.	Prevention approach: Pressure mapping to identify AP and PP of pretibial region in racing wheelchairs in static and dynamic conditions. Prevention approach: Pressure mapping to identify kneeling plate angle AP and PP to inform positioning in racing wheelchair.	Pressure mapping is effective in capturing AP and PP in pretibial region of racing wheelchairs in static and dynamic conditions. Kneeling plate angles significantly correlated with dynamic AP (*r* = 0.45, *p* = 0.08). Kneeling plate angles not significantly correlated with static AP (*r* = 0.35, *p* = 0.18) or static PP (*r* = 0.37, *p* = 0.16).
Tang et al. (2019)	Pressure mapping using pressure sensor Tex pressure mat during three action sequences (still, backward, forward), (still, right, left), (still, small movements)) to determine left and right mean pressure and standard deviations.	Prevention approach: Implementing design modifications to para table tennis wheelchairs to reduce imbalanced seat pressure distribution which can lead to PI. Prevention approach: Pressure mapping to determine left/right seated pressure distribution to reduce seated imbalances.	Pressure mapping was completed prior to modifications. No mapping occurred after the design enhancements were made. There is no indication as to whether the modifications influenced pressure.
Peters et al. (2020)	Pressure mapping using the Boditrak pressure mapping system with wheelchair basketball athletes. Peak pressure index (PPI) and peak pressure gradient (PPG) measured at 4 Hz over 10‐s trial. Variables measured were backrest height, seat dump angle and cushions type (hard foam cushion, soft foam cushion and therapeutic cushion) in static and dynamic conditions.	Prevention approach: Pressure mapping to determine PPI and PPG and its association to seat dump angle and backrest height. Prevention approach: Pressure mapping to determine association between cushion type and PPI/PPG.	Increase in seat dump angle was negatively associated with static PPI (*r* = −0.50, *p* = 0.04) (*r* = −.46, *p* = 0.06) and a moderate negative association with dynamic PPI (*r* = −0.50, *p* = 0.04). No correlation between seat dump angle and PPG (*p* > 0.05). Backrest height was associated with reduced peak pressure in wheelchair basketball athletes. Backrest height showed a moderate negative association with static and dynamic PPI (*rs* = −0.47, *p* = 0.05, *rs* = −0.49, *p* = 0.04). Cushion type was moderately negatively associated with dynamic PPG (*r* = −0.53, *p* = 0.03). No significant relationship to static PPG, PPI and dynamic PPI. Therapeutic cushion use showed a moderately reduced magnitude of PPG compared with harder, rigid cushions.
Ay Yıldız et al. (2025)	Two digital questionnaires administered utilising the attitude scale toward pressure ulcer prevention. 12 semi structured interviews conducted with coaches and athletes.		Athletes reported using non evidence based prevention strategies, including ring sitting pillows and massage to move the blood. Athletes reported having access to nursing while travelling would increase their confidence and contribute to reduce pressure injury severity. Athletes reported having low awareness of health issues and needing increased education on prevention. As athlete experience increases, pressure injuries regress with wound care dressings. Athletes expect coaches to share responsibility of pressure care.

### 3.3 Contextual and environmental factors—Knowledge, guidelines, and recommendations

Two grey literature sources addressed the contextual and environmental domain, providing recommendations relevant to the broader sporting environment in which pressure injury risk occurs. Both were produced in Australia and drew primarily on evidence from non‐sport populations.

The Exercise and Sports Science Australia (ESSA) position statement (2017) on exercise and spinal cord injury (SCI) emphasised proactive consumer engagement through systematic monitoring, individualised equipment and regular use of pressure relieving strategies. The position statement drew on two pressure injury references, neither of which was sport‐specific. The Australian Institute of Sport (AIS) Clinical Practice Guideline: Pressure Injuries and Skin Health in Para Sport (Direen et al., 2025) is the most comprehensive sport contextualised resource currently available covering prevention (equipment, nutrition, and comorbidity management) and wound management with 11.1% of its citations specific to sporting populations. See Table 5, which provides a detailed description of prevention and management strategies identified.

**TABLE 5:** Practice statement and guideline recommendations.

Author(s)	Publishing organisation	Prevention recommendations	Management recommendations
Tweedy et al. (2017)	Exercise and Sports Science Australia (ESSA)	Clear identification of insensate areas via client self‐report Clients use their own cushions when using adaptive equipment where possible/practical Thorough skin checks for red areas following new activities Regular pressure relief during exercise (every 15–30 min) Appropriate protective sports equipment Stretch and passive movements for contracture treatment as it is associated with pressure injury	Nil
Direen et al. (2025)	Australian Institute of Sport (AIS)	General prevention Regular pressure area care and offloading techniques (e.g., forward lean, side lean, and upper body lift) Frequent skin checks, especially bony prominences Use of pressure‐relieving cushions and mattresses Correct wheelchair fitting and posture alignment Avoid friction and shear during transfers Use of appropriate clothing (e.g., breathable, seamless, and non‐slippery fabrics) Avoid massage over bony prominences Medical and lifestyle Optimise management of medical comorbidities (e.g., diabetes and neurological disorders) Smoking cessation and moderation of alcohol consumption Continence management and use of barrier creams Avoid medications that impair wound healing unless essential Maintain hydration and avoid dehydration, especially during travel Nutrition‐based Well‐balanced, nutrient‐rich diet Monitor weight fluctuations and body composition Adequate protein intake (1.5–2 g/kg/day) Include foods rich in zinc and vitamin C Consider fish oil and arginine supplementation if needed Environmental and equipment Monitor environmental conditions (e.g., heat, humidity, and cold) Use custom travel cushions and compression garments during flights Skin checks pre‐ and post‐travel, especially after long flights or transfers Ensure proper fitting of prosthetics, orthoses, and braces Regular review and adjustment of seating and equipment Avoid new shoes or uniforms that may cause pressure Psychosocial and education Build partnerships between athletes and HCPs Educate athletes on pressure care and self‐monitoring Address mental health and social support needs Normalise pressure injury experiences to reduce stigma Training and competition Pressure relief techniques during training (e.g., forward lean, side lean, and upper body lift every 20–30 min) Custom seating and cushions for sport wheelchairs to distribute pressure evenly Regular equipment checks (e.g., tyre inflation and caster alignment) to prevent posture‐related pressure Sport‐specific clothing: Breathable, seamless, non‐slippery fabrics to reduce friction and heat retention Avoid new shoes or uniforms before competition to prevent skin breakdown Equipment fit and function Wheelchair configuration tailored to sport demands and athlete's body morphology Prosthetic fit reviews every 12 months or after body composition changes Sporting equipment scripting to match athlete's functional needs and reduce pressure risk Athlete education and empowerment Self‐monitoring routines for skin health Education on pressure relief and skin care Partnership with HCPs to align sport goals with skin health strategies	Acute injury response Immediate offloading of pressure from affected area Sport‐compatible dressings that do not interfere with performance Photographic documentation and wound tracking during events Multidisciplinary support Team‐based care involving physiotherapists, prosthetists, dietitians, and wound care nurses Referral pathways for infection, pain, or equipment‐related complications Wound care in sport context Dressing selection based on wound stage and sport activity: Lightweight, breathable dressings for training Antimicrobial dressings for infected wounds Debridement techniques adapted to sport schedules and athlete tolerance Pain and infection management Pain control that complies with anti‐doping regulations (e.g., paracetamol and cautious NSAID use) Infection control with topical antimicrobials and systemic antibiotics when needed Recovery and return to sport Nutrition support for healing (e.g., protein, zinc, vitamin C, and arginine) Monitoring for healing progress to guide return‐to‐play decisions Scar management with silicone gels and pressure garments Documentation and communication Clear documentation of injury, treatment, and referrals Medical summaries for travel and competition settings

## 4 DISCUSSION

This scoping review aimed to explore what is currently known about pressure injury prevention and management approaches among individuals with mobility impairments participating in disability sport and the reported effectiveness of identified approaches. Findings revealed that the evidence base is narrow in scope, heavily weighted toward biomechanical studies, and guidance documents are largely built on research conducted outside of sporting contexts. Across the 10 sources, the majority of empirical evidence addressed interface pressure, with almost no investigation of the activity, participation, or contextual dimensions of pressure injury risk: domains central to occupational therapy practice. Addressing effectiveness, this review found no study that directly measured the clinical effectiveness of a pressure injury prevention or management approach in disability sport. All quantitative studies used interface pressure as a proxy outcome measure, an indicator of mechanical load rather than a measure of whether pressure injuries were prevented, reduced in severity, or more rapidly healed. The consistency of this methodological focus across studies in different sports contexts suggests a shared assumption that interface pressure reduction is the most critical element of pressure injury prevention in sport. Sample sizes were small, ranging from 1 to 26 participants, and sport type varied between studies. The predominant methodology was cross‐sectional, which limits the capacity for researchers to understand how pressure injuries may develop over time. Collectively, the evidence base is insufficient to support conclusions about the effectiveness of any specific approach in disability sport.

A lack of research that addresses the factors outside of the biomechanical has important implications for occupational therapists, who require an integrated understanding of how personal, environmental and occupational factors interact to support people with disabilities to maintain skin integrity and enable ongoing sport participation. The International Classification of Functioning, Disability and Health (ICF) provides a lens for understanding the complexity of interaction between body functions, activity demands, participation requirements, and contextual factors that collectively contribute to pressure injury risk in athletes (World Health Organization, 2001). Research included in this review provides a narrow view of the breadth of the complexity of pressure injury aetiology as it exclusively targeted the body functions and structures domain and failed to investigate how activity, participation, and personal/environmental demands contribute to injury risk. Occupational therapists adopt a distinctly holistic perspective, frequently drawing on the ICF framework to understand how an individual's functioning arises from the dynamic interplay of personal, environmental, and occupational factors (McIntyre & Tempest, 2007), making them well positioned to support athletes in pressure injury prevention across all domains.

### 4.1 Biomechanically concentrated evidence base

The distribution of evidence across ICF domains was markedly uneven with seven of eight empirical studies addressing the body functions and structures domain exclusively, examining interface pressure as the primary mechanism of risk (Anderson et al., 2018; Berthold et al., 2013; Darrah et al., 2016; Lewis et al., 2017; J. Peters et al., 2020; Rice et al., 2019; Tang et al., 2019). These studies provide preliminary evidence that equipment configuration and positioning influence interface pressure in sporting contexts; however, sample sizes across all studies were small; no study measured actual pressure injury development as an outcome and the heterogeneity of sports, equipment types, and participants limits cross‐study generalisation.

While increased interface pressure is an important contributor to pressure injury risk, it represents only one component within a range of risk factors (Paquin, Nindorera, et al., 2025). Consequently, research that concentrates solely on interface pressure inevitably overlooks other critical contributors to injury risk, including activity demands, environmental and personal factors, and behavioural practices (Paquin, Lamontagne, & Routhier, 2025). The dominance of engineering and biomedical perspectives in the existing literature (most contributions were from engineers and medical doctors) reflects a disciplinary gap. Occupational therapists routinely analyse the interaction between person, environment, and occupation and are positioned to bring a broader analytical lens to pressure injury management in sports, yet their perspectives are largely absent from the current evidence base.

### 4.2 Gaps in activity, participation, and contextual evidence

Evidence addressing the activity and participation domain was limited to a single study with Ay Yıldız et al. (2025) finding that para‐athletes and coaches had limited and largely non–evidence‐based knowledge of pressure injury prevention. For example, athletes relied primarily on peer experience and strategies that are not recommended in current clinician guidelines (European Pressure Ulcer Advisory Panel et al., 2019). These findings highlight that there may be a gap between the evidence base and the practices of athletes in real sports contexts. Further exploration of how athletes understand and manage pressure injury risk during participation is required.

The absence of activity level evidence is particularly consequential for occupational therapy practice. Occupational therapists are well trained in analysing the specific demands of an occupation, for example, propulsion mechanics, transfer frequency, competitive intensity, positioning requirements, and identifying where these demands intersect with individual risk factors (Law et al., 1996). Without sport‐specific evidence addressing these activity and participation factors, occupational therapists are required to make critical clinical decisions about pressure injury prevention in sport using evidence that was not generated in this context.

The contextual and environmental domain was addressed only through grey literature with both the ESSA position statement (Tweedy et al., 2017) and the AIS Clinical Practice Guideline (Direen et al., 2025); however, both sources drew primarily from non‐sport evidence. As a result, current guidelines provide generalised recommendations that do not account for the contextual factors unique to disability sport, including the performance‐oriented culture of elite sport, the use of specialised adaptive equipment, and the logistical demands of training, competition and travel.

### 4.3 The limits of extrapolation from the general evidence base

Although there is a wealth of research evidence, guidelines and tools for pressure injury prevention and management for the general population (European Pressure Ulcer Advisory Panel et al., 2019), participation in sports is a distinct context and requires context‐specific evidence to guide decision making. For example, previous research has suggested that some athletes may prioritise performance, competition availability, and training continuity, which can incentivise risk‐taking behaviours such as reduced adherence to off‐loading, training through early skin warning signs, or using equipment that favours performance over protection (Fagher et al., 2016). Elite athletes also may have different motivators for participation than amateurs, which can influence their propensity for risk and adherence to management strategies (Osmotherly et al., 2021). However, these findings are based on limited evidence and should be interpreted cautiously as one possible contributor to behavioural risk rather than an established pattern. The implication of this research is that athletes may engage in risk‐taking behaviours beyond those of their non‐athlete peers, and specific strategies are required to support these athletes to balance performance with risk management.

Adaptive equipment used in disability sports has no direct comparator in typical clinical populations. This equipment relies on custom geometries and configurations purpose built for performance, which can influence injury risk (Haydon et al., 2016). Custom moulded carbon fibre seating and specialised strapping systems introduce unique and potentially higher interface pressures (Maeda et al., 2021), a finding echoed by Anderson et al. (2018) who observed significantly higher pressures in adaptive equipment compared with daily wheelchairs. These differences may limit the extent to which general clinical evidence can be confidently applied to sporting populations without empirical validation.

This review found that, considering the potentially unique demands of sport, there was insufficient empirical evidence to make strong recommendations on prevention and management strategies for pressure injuries in sports. Only eight empirical studies were identified that specifically examined pressure injuries in sport, which is in stark contrast to the substantial body of research in non‐sports settings (Pott et al., 2023; Reddy et al., 2006). This is further evidenced when examining the citations used in the position statements and guidelines identified in this review. In the ‘Clinical Practice Guideline: Pressure Injuries and Skin Health in Para Sport’ (2025), 11.1% of citations were specific to people experiencing pressure injuries while participating in sport. Further, of the 71 articles referenced in the ‘Exercise and sports science Australia (ESSA) position statement on exercise and spinal cord injury’ (Tweedy et al., 2017), two were pressure injury related; however, it did not describe recommendations specific to sport. ESSA's position statement has been widely cited in disability sports contexts (Pelletier, 2023; Pinheiro et al., 2024; Rozenstoka et al., 2025), but it does not contribute population‐specific research, with recommendations instead being extrapolated from general population pressure injury literature. Extrapolating from general literature is generally accepted when the mechanisms of risk are shared and the specific population is under researched; however, extrapolation requires careful boundaries and an awareness that research may underestimate risk (U. Peters, Sherling, & Chin‐Yee, 2024).

When guidelines rely on evidence derived from non‐athlete populations, the recommendations may not reflect the unique biomechanical, environmental and behavioural risk factors present in disability sport. Clear guidance driven by context specific research has the potential to reduce inconsistent and misaligned interventions (Serafin et al., 2025). Strengthening the empirical evidence base will support future guideline development and equip clinicians with the resources needed to provide evidence‐based assessment and intervention.

### 4.4 The occupational therapy contribution

To the authors' knowledge, no research has examined pressure injury prevention and management in sports through the lens of occupational therapy practice. Despite the absence of occupational therapy specific research in the included literature, emerging evidence points to the contribution occupational therapy can make in disability sport. A recent scoping review by Bulan et al. (2025) found that occupational therapy interventions in sports consistently reported positive outcomes; for example, wheelchair rugby athletes reported significantly improved safety and performance after assessment and modification of their equipment. In one study of para‐athletes, occupational therapy was consistently cited as being a key facilitator to participation, tailoring intervention to meet athletes' unique needs and ensuring satisfaction with prescribed assistive technology (Silva & Alves, 2020). These findings, while limited in scope, suggest that an occupational therapy contribution to pressure injury prevention in sport, one that moves beyond biomechanical assessment to encompass occupational analysis, person‐environment‐occupation fit, and athlete‐centred goal setting, has the potential to meaningfully improve both safety and participation outcomes.

This scoping review was subject to several methodological limitations. First, the inclusion criteria restricted the review to studies published in English, potentially excluding relevant research. A comprehensive search strategy was developed in conjunction with a librarian; however, it remains possible that relevant published studies and grey literature were inadvertently excluded. Although the JBI review methodology was used, scoping reviews do not employ the rigorous methodological protocols of a systematic review and are unable to draw specific conclusions and recommendations; rather, this review provides an overview of the literature. Despite these limitations, this scoping review's strength lies in it being, to the authors' knowledge, the first of its kind to explore pressure injury prevention and management specific to disability sports. With the growth of disability sports, this review provides a foundation for future research in the area.

## 5 CONCLUSION

Sport participation is a meaningful occupation, and pressure injuries represent a potential source of occupational disruption and deprivation for athletes whose identity, wellbeing and community connection are tied to their sport. Occupational therapists are uniquely positioned to address this risk through an occupation‐centred perspective that extends beyond equipment assessment to encompass the dynamic interaction between the athlete, their environment, and the demands of their sport.

This scoping review identified that evidence addressing pressure injury prevention and management in disability sports is limited in scope, biomechanically concentrated, and largely extrapolated from non‐sport populations. Across the included sources, evidence was heavily weighted toward body functions and structures, with minimal investigation of the activity, participation, and contextual dimensions of risk that are central to occupational therapy practice. This highlights the need for sport‐specific, holistic research that captures the lived realities of athletes, occupational demands, and complexities of participating in disability sport. To support clinical decision making and the development of relevant, evidence‐informed guidelines, research must prioritise sport‐specific investigation that incorporates the perspectives of athletes, coaches and allied health professionals. Building the evidence base is essential to support occupational therapists in delivering effective, contextually appropriate interventions that enable safe entry and ongoing participation in disability sport.

## AUTHOR CONTRIBUTIONS

**Callum Parker**: Conceptualization (lead); writing—original draft (lead); writing—review and editing (lead). **Sjaan Gomersall:** Conceptualisation (supporting); writing—review and editing (supporting). **Emma Beckman:** Writing—review and editing (supporting). **Sean Tweedy:** Writing—review and editing (supporting). **Jessica Hill:** Conceptualisation (supporting); writing—review and editing (supporting).

## CONFLICT OF INTEREST STATEMENT

The authors have no conflict of interest to declare.

## ETHICS STATEMENT

Not applicable.

## DECLARATION OF USE OF ARTIFICIAL INTELLIGENCE

Microsoft Co‐Pilot was used during drafting for improving clarity/flow of written text. The output was checked, refined, and confirmed by the authors before inclusion.

## CONSENT FOR PUBLICATION

Not applicable.

## DECLARATIONS

No declarations.

## Data Availability

Not applicable.
